# A two-stage hybrid gene selection algorithm combined with machine learning models to predict the rupture status in intracranial aneurysms

**DOI:** 10.3389/fnins.2022.1034971

**Published:** 2022-10-20

**Authors:** Qingqing Li, Peipei Wang, Jinlong Yuan, Yunfeng Zhou, Yaxin Mei, Mingquan Ye

**Affiliations:** ^1^School of Medical Information, Wannan Medical College, Wuhu, Anhui, China; ^2^Research Center of Health Big Data Mining and Applications, Wannan Medical College, Wuhu, Anhui, China; ^3^Department of Neurosurgery, Yijishan Hospital of Wannan Medical College, Wannan Medical College, Wuhu, Anhui, China; ^4^Department of Radiology, Yijishan Hospital of Wannan Medical College, Wannan Medical College, Wuhu, Anhui, China

**Keywords:** intracranial aneurysms, rupture status, gene selection, machine learning, FCBF-MLP-PSO, informative genes

## Abstract

An IA is an abnormal swelling of cerebral vessels, and a subset of these IAs can rupture causing aneurysmal subarachnoid hemorrhage (aSAH), often resulting in death or severe disability. Few studies have used an appropriate method of feature selection combined with machine learning by analyzing transcriptomic sequencing data to identify new molecular biomarkers. Following gene ontology (GO) and enrichment analysis, we found that the distinct status of IAs could lead to differential innate immune responses using all 913 differentially expressed genes, and considering that there are numerous irrelevant and redundant genes, we propose a mixed filter- and wrapper-based feature selection. First, we used the Fast Correlation-Based Filter (FCBF) algorithm to filter a large number of irrelevant and redundant genes in the raw dataset, and then used the wrapper feature selection method based on the he Multi-layer Perceptron (MLP) neural network and the Particle Swarm Optimization (PSO), accuracy (ACC) and mean square error (MSE) were then used as the evaluation criteria. Finally, we constructed a novel 10-gene signature (YIPF1, RAB32, WDR62, ANPEP, LRRCC1, AADAC, GZMK, WBP2NL, PBX1, and TOR1B) by the proposed two-stage hybrid algorithm FCBF-MLP-PSO and used different machine learning models to predict the rupture status in IAs. The highest ACC value increased from 0.817 to 0.919 (12.5% increase), the highest area under ROC curve (AUC) value increased from 0.87 to 0.94 (8.0% increase), and all evaluation metrics improved by approximately 10% after being processed by our proposed gene selection algorithm. Therefore, these 10 informative genes used to predict rupture status of IAs can be used as complements to imaging examinations in the clinic, meanwhile, this selected gene signature also provides new targets and approaches for the treatment of ruptured IAs.

## Introduction

An intracranial aneurysm (IA) is an abnormal swelling of cerebral vessels, which can occur without causing any symptoms (Pontes et al., 2021). A subset of these IAs can rupture, causing aneurysmal subarachnoid hemorrhage (aSAH), often resulting in death or severe disability (Tawk et al., 2021). In general, assessing the number of subarachnoid hemorrhages requires imaging studies such as *trans-*cranial Doppler, computed tomography (CT), and magnetic resonance imaging (MRI) which are sometimes difficult to obtain especially in complicated patients and are technically demanding for physicians (Rose, 2011). With the rapid growth of RNA-sequencing (RNA-seq) technologies, massive sequencing data have been produced in the area of tumor research. In the field of IA research, evaluating the status of IAs by transcriptomic profiling has also become a hotspot, with several studies focusing on mining the molecular biomarkers from transcriptomic data to predict the status of an IA (Gao et al., 2020; Poppenberg et al., 2020). To date, few studies have used the method of feature selection combined with machine learning in this area; however, in the early stages of subarachnoid hemorrhage, using the appropriate method to distinguish quantity controlled molecular markers can help to precisely predict the status of an IA and may provide new therapeutic targets.

Intracranial aneurysm transcriptomic sequencing data, similar to gene expression data from other tumors, often have the properties of a small sample size and high dimensional features, large amounts of redundant or unnecessary features may not only result in misdiagnosis and failure to diagnose but can also be time-consuming and reduce the effectiveness of the categorization (Chen et al., 2016; Xiong et al., 2021). To better acquire effective information from gene expression data, currently, our objective is to successfully reduce the feature dimensionality and obtain an informative subset of genes with the best categorization performance. With traditional statistical methods, multiple genes from the same pathway are selected, as genes in the same pathway tend to have the same or similar expression pattern, which can lead to the introduction of a particular set of gene signatures involved in one particular biological process by overrepresentation and thus introduce considerable redundancy (Perscheid, 2021). Machine learning models have unique advantages in addressing issues such as clustering, classification, and regression of high-dimensional biological multi-omics data (Camacho et al., 2018), feature selection is a key step of machine learning in the preprocessing of gene expression sequencing data, which is beneficial to precision medicine, can help discover disease mechanisms and reduce the cost of clinical diagnosis by finding the optimal set of features based on the performance of classification models (Chen et al., 2021).

When managing RNA-seq data and gene microarray data, the feature selection process that before machine learning modeling was commonly referred to as informative gene selection. The purpose of gene selection is to eliminate completely unrelated and noise features, weak correlation and redundancy features, and to filter out strong correlation features related to modeling. The optimal subset of features obtained by feature selection should theoretically make the model run faster, with higher model performance, and unlike feature extraction, the value of the eigenvalues in the data does not change after feature selection. Depending on the way the subset of features is evaluated, gene feature selection methods can be classified as filter-based methods, wrapper-based methods and embedded-based methods, as well as hybrid-based methods and ensemble-based methods, which have been popular in recent years (Jain et al., 2018; Ye et al., 2019). Without taking into account any particular learning algorithm, filter methods eliminate genes with minimal information based on the statistical properties of variables, and mainly include correlation-based feature selection, the Markov blanket filter method, and the mutual information-based methods (Yu and Liu, 2003; Tang et al., 2018). Among them, the Fast Correlation-Based Filter (FCBF) is a filtering solution based on the two features through the Symmetrical Uncertainty (SU) method as a measure, which have been widely used in high-dimensional gene expression profiles (Lei and Liu, 2003). Wrapper methods can obtain a relatively small subset of genes with better classification through performance by evaluating the performance of the predefined learning algorithm (Huang et al., 2020). Informative gene selection and the training procedure are conducted concurrently using embedded methods, which incorporate gene selection into the learning algorithm (Medjahed et al., 2017).

In this study, we propose a two-stage hybrid algorithm FCBF-Multi-layer Perceptron (MLP)-Particle Swarm Optimization (PSO), that is, a mixed filter- and wrapper-based feature selection. Firstly, using the FCBF algorithm to filter a large number of irrelevant and redundant genes in the raw dataset, and then using the wrapper feature selection method based on the MLP neural network and PSO, MLP as a classifier, the PSO algorithm as the search strategy, and using accuracy (accuracy) and mean square error (MSE) as the evaluation criteria, the feature set with fewer variable numbers and high classification ACC is finally obtained as the optimal feature subset. Our findings demonstrated that the proposed method can improve classification performance while also obtaining a smaller subset of informative genes, which will benefit the mining of IA rupture-related biomarkers.

## Materials and methods

### Data collection

The gene expression omnibus (GEO) datasets^1^ are maintained by NCBI to store gene expression profiles by RT-PCR, high-throughput sequencing, microarray and so on. We downloaded four gene expression datasets from the GEO, GSE13353 series on the GPL570 platform (Affymetrix Human Genome U133 Plus 2.0 Array) (Kurki et al., 2011), GSE15629 on the GPL6244 platform (Affymetrix Human Gene 1.0 ST Array) (Pera et al., 2010), GSE54083 on the GPL4133 platform (Agilent-014850 Whole Human Genome Microarray 4x44K G4112F) (Nakaoka et al., 2014), and GSE122897 on the GPL16791 platform (Illumina HiSeq 2500) (Kleinloog et al., 2016), since when we searched the GEO database and found that only these four GSE gene expression datasets meet our requirements that contained clear information on whether IAs ruptured or not. Data from different platforms were normalized and centered before the “sva” R package was used to remove the batch effects. After removing negative controls that were not IA, samples with explicit status of ruptured and unruptured aneurysms were retained. Finally, 88 samples entered subsequent analysis, including 48 ruptured and 40 unruptured samples of IA. The ruptured and unruptured groups were labeled 1 and 0, respectively. 0 and 1 were also used as target labels for binary samples by the following feature selection and machine learning algorithms.

### Recognition and enrichment analysis of differentially expressed genes

After data had been downloaded and integrated, R package “limma” (Ritchie et al., 2015) suitable for both RNA-seq and microarray studies was used to generate DEGs between the two groups based on linear models. *P*-value < 0.05 and the | Fold-change| > 1.5 were the requirements for DEG significance. R package “pheatmap” was used to display the heatmap plot and visualize the results of differential expression analysis. Then, non-redundant gene biological terms in a functionally grouped network were clustered and visualized using the Cytoscape (version 3.8.0) desktop application and the “ClueGO” plug-in. The Gene Ontology (GO) and Kyoto Encyclopedia of Genes and Genomes (KEGG) pathway enrichments of up-regulated and down-regulated DEGs were separately performed by web-based Metascape (Zhou et al., 2019).

### Evaluation of immune cell infiltration

TIMER 2.0^2^ was used to provide a robust estimation of immune infiltration levels in each sample of IA (Li et al., 2020), algorithms including TIMER, CIBERSORT, quanTIseq, xCell, MCP-counter and EPIC were all implemented. The significance criterion was *P*-value < 0.05 by two-tailed *t*-test, the expression profiles of immune cells with significant differences between groups in each sample are shown by heatmaps, and the overall profiles of each group are shown by box plots.

### The proposed two-stage hybrid feature selection algorithm

#### Related theory

(1) The FCBF is a filtering solution based on fast correlations, as proposed by Lei and Liu (2003). The core idea of the algorithm is to measure the correlation of the two features through the SU method as a measure, the SU value of each feature *g_i* vs. the category C is calculated as$S\,U\,\left(X,Y\right)=2\,\left[\frac{H\,\left(X\right)-H\,\left(X|Y\right)}{H\,\left(X\right)+H\,\left(Y\right)}\right]$, namely (*g*_*i*_,*C*), where *H(X)* represents the information entropy, and *H*(*X*|*Y*) represents conditional entropy. The filter-based feature selection method based on FCBF is suitable for solving the feature selection problem of large-scale data, and can effectively delete redundant and irrelevant features in high-dimensional data. However, because the filter-based feature selection method is separated from the evaluation strategy of the learning algorithm in the process of gene subset selection, it is difficult to determine whether the selected feature subset can make the classification algorithm achieve the best performance.

(2) The MLP is an artificial neural network that tends to structure. This algorithm maps a set of input vectors to a set of output vectors by including a network structure with an input layer, hidden layer and output layer (Yang et al., 2009). The output of the MLP neural network can be expressed as $f_{k}\,\left(d\right)=\frac{1}{1+e\,x\,p\,\left[-g_{k}\,\left(d\right)\right]}$, where *g*_*k*_(*d*) is the weighted sum of the hidden layer nodes, *k* = 1,2,⋯,*m*; *d* is the eigenvalue vector of the MLP neural network, with *d* = (*d*_1_,*d*_2_,⋯,*d*_*i*_). The MLP based wrapped feature selection method selects feature subsets that are more relevant to the classification algorithm than the filtered feature selection algorithm, which is not conducive to solving the difficulties of feature selection and classification caused by the complex sample and feature distribution characteristics of the bionomic data, and has low computational efficiency in high-dimensional data processing.

(3) The PSO is a group intelligent search algorithm that simulates birds feeding on food in nature (Rostami et al., 2020). The PSO algorithm places a population of particles in the *D*-dimensional search space and evaluates the fitness of each particle. In the PSO algorithm, the *i-*th particle can update its next-generation position and flight speed with the following two formulations:


$$x_{i}^{t+1}=x_{i}\,\left(t\right)+v_{i}^{t+1}$$

$$v_{i}^{t+1}=\omega^{*}\,\,v_{i}^{t}+c_{1}^{*}\,\,r\,a\,n\,d_{1}^{*}\,\left(p\,b\,e\,s\,t_{i}-x_{i}^{t}\right)+c_{2}^{*}\,\,r\,a\,n\,d_{2}^{*}\,\left(g\,b\,e\,s\,t_{i}-x_{i}^{t}\right)$$

where *t* represents the current number of iterations, the $x_{i}^{t+1}$ and $v_{i}^{t+1}$ indicate the position and flight speed of the particle under the iterations *t* + 1, respectively;ω represents the inertia weight used to adjust the effect of the particle velocity of the previous generation on the current particle velocity; the factors *c_1* and *c_2* indicate the acceleration coefficient, representing the cognitive learning factors and the social learning factors, respectively, and they are used to adjust the contribution of the individual optimal position *pbest* and the global optimal position *gbest* to the particles, which is usually set to 2; the *rand*_1_ and *rand*_2_ are represented as random numbers in the [0,1] range.

The main purpose of the two-stage hybrid feature selection method (FCBF-MLP-PSO) is to overcome the shortcomings of the existing filter-based or wrapper-based gene feature selection methods. First, the FCBF filtering feature selection method is used to quickly remove redundant features and generate candidate feature subsets, which can significantly reduce the computational complexity of feature selection for high-dimensional data. Then, the wrapped feature selection method based on MLP is adopted, and an improved particle swarm search strategy is introduced for secondary feature selection. The combined feature subset with strong discrimination ability is selected to overcome the problems of combined features being deleted by mistake and the deviation between the feature evaluation results and the final classification algorithm, thus significantly improving the classification ACC of gene expression in related diseases.

#### Steps of the proposed hybrid gene selection algorithm

This study proposed a mixed filter- and wrapper-based feature selection algorithm FCBF-MLP-PSO, the proposed hybrid algorithm includes the following steps:

Step 1: The FCBF algorithm was used to filter a large number of irrelevant and redundant genes in the original dataset through SU.
Step 2: The wrapper feature selection method based on the MLP neural network and PSO was used, MLP as a classifier, and PSO algorithm as the search strategy, using ACC and MSE as the evaluation criteria, to finally obtain the feature set with fewer variable numbers and high classification ACC as the optimal feature subset.
Step 3: Several classification algorithms were used to evaluate the effectiveness of gene subsets, including eXtreme Gradient Boosting (XGBoost), LightGBM, Random Forest (RF), Extra Tree (ET), Gaussian NB, K-nearest neighbors (KNN), Logistic Regression (LR), Decision Tree (DT), Support Vector Machine (SVM), and Linear Discriminant Analysis (LDA). Each algorithm used the comprehensive evaluation criteria of classification ACC, recall, F1 value, area under ROC curve (AUC) and confusion matrix by 10-fold cross-validation.

**FIGURE 1:**
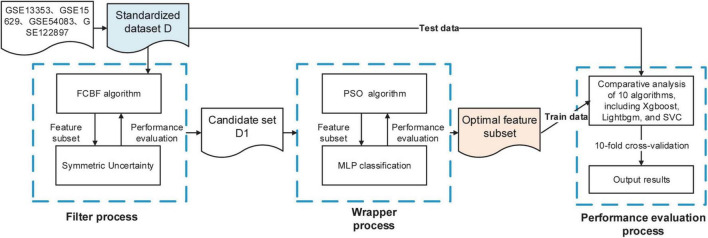
The experimental flow of the proposed hybrid gene selection algorithm FCBF-MLP-PSO.

The WEKA 3.9.6 software platform and Python 3.8 were used to complete the aforementioned experiments. The experimental flow of the gene selection algorithm suggested in this research is shown in Figure 1. The method can effectively decrease the size of the raw gene sets, obtain fewer genes, and have higher classification ACC.

## Results

### Data description

After removing negative controls that were not IA and samples with vague status of ruptured and unruptured aneurysms, 88 samples subjected to debatching and normalization processing from four GEO datasets including 48 ruptured and 40 unruptured samples of IA were used in this study, and the details of each dataset are illustrated in Table 1. The sample size was not large, but the number of features was large; therefore, we need to solve the binary classification problem of high-dimensional small samples. In order to control the quality of batch effect correction, we used two dimensionality reduction methods, including Principal Component Analysis (PCA) and t-Distributed Stochastic Neighbor Embedding (t-SNE), to visualize the clustering of all samples from 4 different GSE datasets before and after adjustment of the batch effects (Supplementary Figure 1). Supplementary Figures 1A,B demonstrated that samples from different GSE datasets clearly clustered together before batch effect correction. While Supplementary Figures 1C,D showed that after de batch effect adjustment, the batch effect was eliminated and all samples from 4 different GSE datasets were almost evenly dispersed.

**TABLE 1:** Description of GEO datasets used in this research.

GEO number	Platform	No. of total	No. of ruptured	No. of unruptured	PMID
GSE13353	GPL570	19	11	8	21336216
GSE15629	GPL6244	14	8	6	20044533
GSE54083	GPL4133	13	8	5	24938844
GSE122897	GPL16791	42	21	21	27026628
	Total	**88**	**48**	**40**	

Bold values represent the average performance.

### Differentially expressed mRNAs between ruptured and unruptured intracranial aneurysms

**FIGURE 2:**
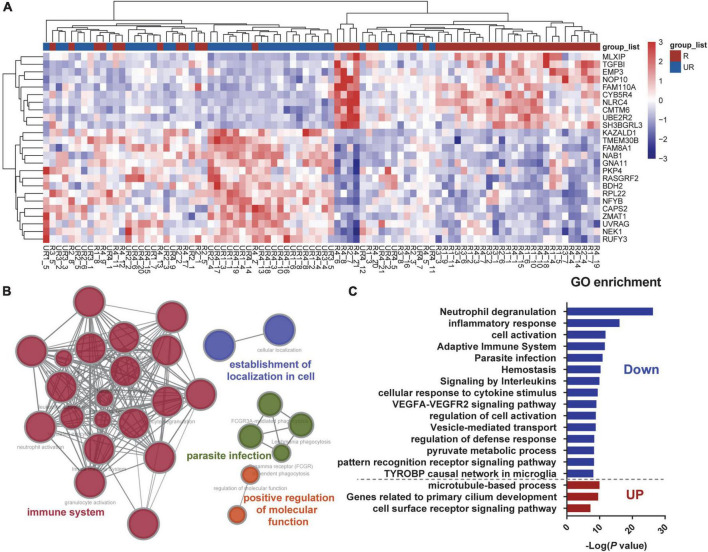
Differentially expressed mRNAs between ruptured and unruptured intracranial aneurysms. **(A)** Heatmap plot presents the first 25 genes exhibiting the most significant fold change across all samples, and the ruptured and unruptured aneurysm samples are hierarchically clustered. **(B)** Gene Ontology (GO) enrichment of the differentially expressed mRNAs between the two groups. **(C)** Pathway enrichments of up-regulated and down-regulated DEGs were analyzed separately.

Gene expression data have many redundant features. To improve classification efficiency and to identify distinctive subgroup-specific patterns with different status of ruptured and unruptured aneurysms, we first conducted an analysis of DEGs. A total of 913 mRNAs passed the threshold screening, including 394 up-regulated genes and 519 down-regulated genes. The heatmap selects the first 25 genes exhibiting the most significant fold change for presentation (Figure 2A), and from the results of hierarchical clustering, the ruptured and unruptured aneurysms can still be separated into two distinct categories even if the data were derived from different batches and different platforms, indicating that the ruptured aneurysms can affect the gene expression patterns of the aneurysms and that our data preprocessing section is feasible. GO analyses using these 913 genes were further conducted and showed that these differentially expressed mRNAs were predominantly enriched in biological processes related to the immune system (Figure 2B). When enrichment analysis was performed separately for up-regulated and down-regulated genes (ruptured vs. unruptured IA), we found that the majority of the GO terms were contributed by down-regulated genes, the most significant of which were neutrophil degranulation and inflammatory response (Figure 2C). These results illustrate that ruptured and unruptured aneurysms are clearly distinguished at the transcriptional level, and these distinctions are closely related to the function of the immune system, with the ruptured aneurysm group exhibiting a marked down-regulation of immune-related genes. The deletion of two sets of genes with similar expression patterns, which reduces the number of features from tens of thousands to 913, can also effectively improve the efficiency of subsequent feature selection.

### Differential immunological characteristics between ruptured and unruptured intracranial aneurysms

**FIGURE 3:**
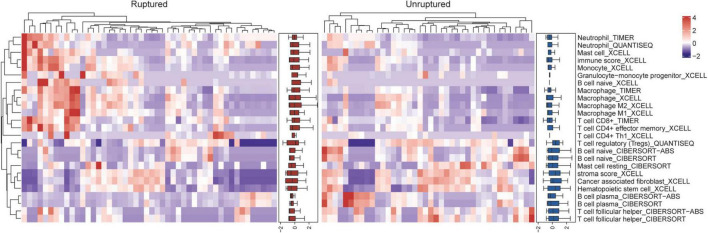
Differential immunological characteristics between ruptured and unruptured intracranial aneurysms.

Considering that the GO terms enriched in neutrophil degranulation and inflammatory response were the most significant in the above results, we speculate that key factors in aneurysm rupture are associated with innate immunity. Multiple algorithms including TIMER, CIBERSORT, quanTIseq, xCell, MCP-counter and EPIC were used to estimate the abundance of tissue-infiltrating immune subpopulations in ruptured aneurysms and unruptured aneurysms, and the differential immunological characteristics between the two groups are shown in Figure 3. We found that most innate immune cells (such as macrophages, monocytes, neutrophils) were highly infiltrated in ruptured IAs, and adaptive immune cells (such as CD4^+^ T cells, CD8^+^ T cells, and B cells) were highly infiltrated in unruptured IAs. These results showed that the distinct status of IAs could lead to differential immune cell composition, thereby influencing the immune responses.

### Machine learning-based prediction of rupture status in intracranial aneurysms

**FIGURE 4:**
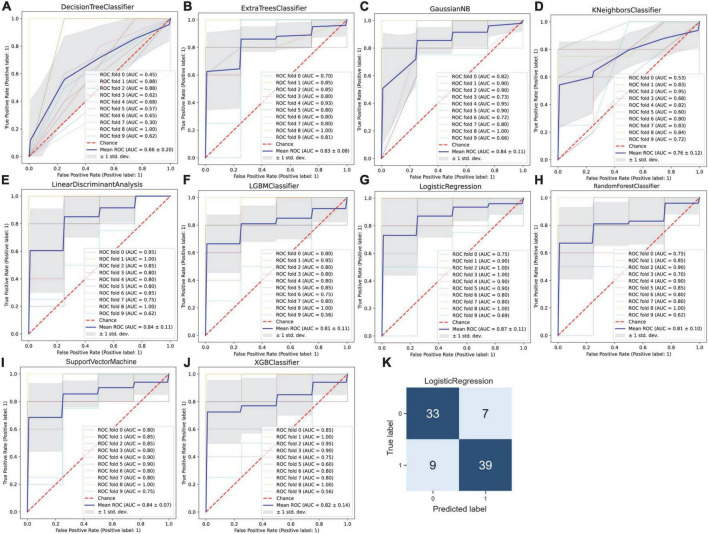
Machine learning-based prediction of rupture status in intracranial aneurysms. **(A)** Decision tree, **(B)** extra tree, **(C)** gaussian NB, **(D)** K-nearest neighbor, **(E)** linear discriminant analysis, **(F)** LightGBM, **(G)** logistic regression, **(H)** random forest, **(I)** support vector machine, and **(J)** XGBoost classifiers were used to establish the classification model. The receiver operating characteristic (ROC) curve was drawn after 10-fold cross-validation for each model, and the mean area under the ROC curve (AUC) was calculated, the mean AUC was combined with the standard deviation (SD). **(K)** The confusion matrix for the logistic regression model with the highest accuracy.

In order to accurately predict whether an IA is ruptured using the integrated gene expression data, we attempted to develop an effective classification model that would distinguish ruptured cases from unruptured cases. Based on the 913 DEGs screened out above as features or modeling, 10 types of machine learning classification algorithms, including XGBoost, LightGBM, RF, ET, Gaussian NB, KNN, LR, DT, SVM, and LDA were used to establish the classification model. The evaluation indicators including ACC, recall, precision, F1 value, and mean AUC by 10-fold cross-validation were calculated and are listed in Table 2. The average ACC of all 10 models was 0.752, average recall was 0.732, average precision was 0.812, average F1 score was 0.753 and the average AUC of all models was 0.808. In addition, the ROC curve with 10-fold cross-validation (Figures 4A–J) and the confusion matrix (Figure 4K) for the LR model with the highest ACC and AUC (ACC = 0.817, AUC = 0.87) was plotted. As shown, the performance of these classifiers was reasonable at this point.

**TABLE 2:** Classification performance tests before gene selection.

Model name	Accuracy	Recall	Precision	F1	AUC
XGBClassifier	0.772	0.730	0.856	0.763	0.82 ± 0.14
LGBMClassifier	0.714	0.750	0.763	0.732	0.81 ± 0.11
RandomForestClassifier	0.763	0.750	0.802	0.749	0.81 ± 0.10
ExtraTreesClassifier	0.758	0.765	0.787	0.758	0.83 ± 0.08
GaussianNB	0.817	0.745	0.885	0.804	0.84 ± 0.11
KNeighborsClassifier	0.713	0.680	0.767	0.713	0.76 ± 0.12
LogisticRegression	0.817	0.815	0.858	0.826	0.87 ± 0.11
DecisionTreeClassifier	0.639	0.675	0.743	0.715	0.66 ± 0.20
SupportVectorMachineClassifier	0.794	0.725	0.895	0.777	0.84 ± 0.07
LinearDiscriminantAnalysis	0.736	0.680	0.752	0.691	0.84 ± 0.11
Mean	**0.752**	**0.732**	**0.812**	**0.753**	**0.808**

Bold values represent the average performance.

### Key gene identification for better prediction of rupture status in intracranial aneurysms by the proposed hybrid gene selection algorithm

While the model prediction performance described above is acceptable, among the 913 genes, there are still many redundant and irrelevant features, which may lead to over-fitting of classification models, and still a large number of genes will be difficult to apply in clinical practice. In order to improve the generalization ability of classifiers and the feasibility of clinical application, we proposed a mixed filter- and wrapper-based feature selection algorithm FCBF-MLP-PSO. The FCBF algorithm was first used, and irrelevant and redundant genes were eliminated to obtain a set of 42 candidate features ranked by their importance to category (Supplementary Figure 2). Then genes were screened by MLP-PSO, and a valid subset of 10 features was selected with better ACC and MSE evaluation criteria from the set of candidate features. Finally, YIPF1, RAB32, WDR62, ANPEP, LRRCC1, AADAC, GZMK, WBP2NL, PBX1, and TOR1B containing a total of 10 effective feature subsets were determined.

**FIGURE 5:**
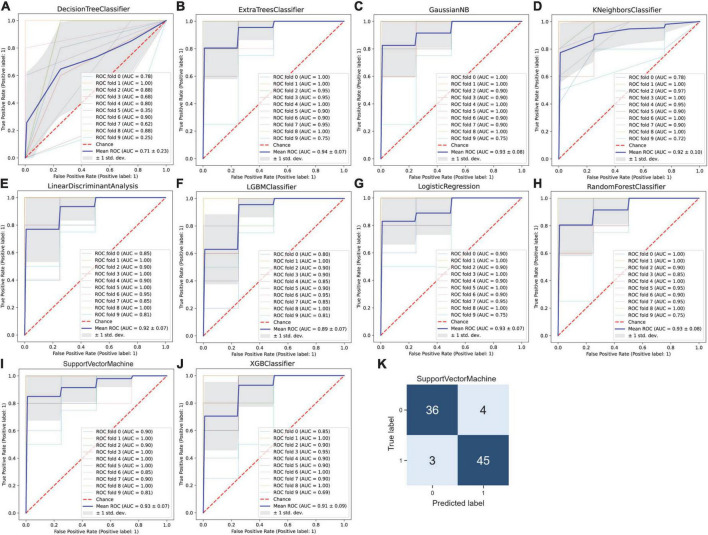
Machine learning-based prediction of rupture status in intracranial aneurysms after gene selection by the proposed hybrid gene selection algorithm. **(A)** Decision tree, **(B)** extra tree, **(C)** gaussian NB, **(D)** K-nearest neighbor, **(E)** linear discriminant analysis, **(F)** LightGBM, **(G)** logistic regression, **(H)** random forest, **(I)** support vector machine, and **(J)** XGBoost classifiers were used to establish the classification model. The receiver operating characteristic (ROC) curve was drawn after 10-fold cross-validation for each model, and the mean area under the ROC curve (AUC) was calculated, the mean AUC was combined with the standard deviation (SD). **(K)** The confusion matrix for the support vector machine model with the highest ACC.

Next, the selected 10 features were fed into the 10 types of different machine learning classification predictive models. The results showed that when using the hybrid gene selection algorithm proposed above, all evaluation indicators including ACC, recall, precision, F1 value, and mean AUC by 10-fold cross-validation, were improved (Table 3). For example, the average ACC value increased from 0.752 to 0.840 (11.7% increase), and the average AUC value increased from 0.808 to 0.901 (11.5% increase). The highest ACC value increased from 0.817 to 0.919 (12.5% increase), the highest AUC value increased from 0.87 to 0.94 (8.0% increase). In addition, the ROC curve with 10-fold cross-validation (Figures 5A–J) and the confusion matrix (Figure 5K) for SVM classifier with the highest ACC was plotted. The confusion matrix also showed that the predicted label matched the true label better, as shown by the larger number of upper left and lower right diagonals, and cumulatively there were only seven cases of sample prediction error. These results indicated that the deletion of redundant and irrelevant features could improve the model’s ACC, whereas reduced features have greater implications for clinical diagnosis.

**TABLE 3:** Classification performance tests after gene selection by the proposed hybrid algorithm.

Model name	Accuracy	Recall	Precision	F1	AUC
XGBClassifier	0.815	0.850	0.815	0.827	0.91 ± 0.09
LGBMClassifier	0.830	0.815	0.868	0.837	0.89 ± 0.07
RandomForestClassifier	0.831	0.825	0.826	0.836	0.93 ± 0.08
ExtraTreesClassifier	0.874	0.855	0.927	0.868	0.94 ± 0.07
GaussianNB	0.850	0.810	0.902	0.845	0.93 ± 0.08
KNeighborsClassifier	0.840	0.79	0.915	0.835	0.92 ± 0.10
LogisticRegression	0.872	0.895	0.887	0.884	0.93 ± 0.07
DecisionTreeClassifier	0.690	0.685	0.798	0.757	0.71 ± 0.23
SupportVectorMachineClassifier	0.919	0.940	0.930	0.929	0.93 ± 0.07
LinearDiscriminantAnalysis	0.874	0.895	0.882	0.883	0.92 ± 0.07
Mean	**0.840**	**0.836**	**0.875**	**0.850**	**0.901**

Bold values represent the average performance.

### Distribution of the selected 10 informative genes across samples between the two groups

**FIGURE 6:**
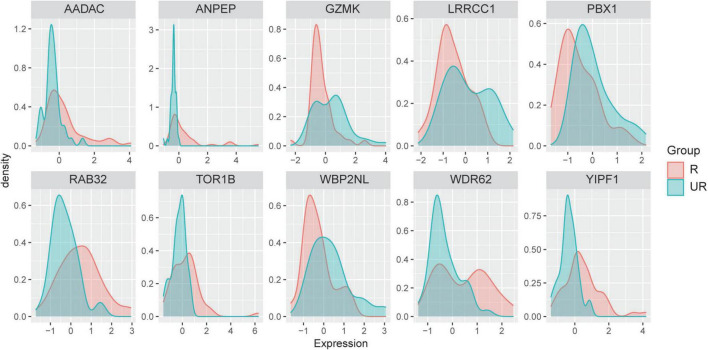
Distribution of the differential expression of the selected 10 informative genes across samples between ruptured and unruptured intracranial aneurysms.

To explore the relationship between the 10 informative genes selected by our proposed hybrid gene selection algorithm and the grouping of ruptured and unruptured IAs, we analyzed the distribution of the differential expression of the selected 10 informative genes across samples. From the distribution plot, it can be seen that the expression pattern between the ruptured group and unruptured group was significantly different (Figure 6), as the peaks of the two groups in the distribution plots were completely inconsistent. We used the normalized and the batch effect processed data when performing feature selection as well as classifier prediction, and for a better view of the roles of these 10 informative genes we additionally confirmed each gene expression per sample in the raw four GSE datasets. Violin plots were used to present the total dataset and boxplots were used to present individual raw GSE datasets. Supplementary Figure 3 shows that YIPF1, RAB32, WDR62, ANPEP, AADAC, and TOR1B were up-regulated in ruptured IAs, while GZMK, LRRCC1, PBX1, and WBP2NL were down-regulated to some extent, indicating that YIPF1, RAB32, WDR62, ANPEP, AADAC, and TOR1B are risk factors, while the others are protective factors. Although the expression of each informative gene in the total dataset was significantly different between the ruptured and unruptured groups, some genes did not reach statistical significance in a certain GSE due to the small sample size. However, the expression pattern differences of these two groups can still be observed in each GSE dataset.

## Discussion

Several studies have investigated the differences in gene expression in IAs vs. normal artery tissues to identify key genes and pathways implicated in the formation of aneurysms. For example, an 18-gene signature distinguished the presence of unruptured IAs with an area under the ROC curve of 0.91 by the SVM model using whole blood transcriptome (Poppenberg et al., 2020). Using the method of taking an intersection after statistical analysis of several GEO databases, an 11-gene signature involving the leukocyte *trans-*endothelial migration pathway has been determined (Gao et al., 2020). However, studies comparing ruptured vs. unruptured aneurysms are relatively few. Using imaging techniques and meta-analysis can accurately assess whether IAs will rupture (Etminan and Rinkel, 2016). Despite this, the exact molecular mechanisms that ultimately cause aneurysm rupture are still uncertain, and the molecular biomarkers involved in the process of IA rupture have yet to be identified.

The use of differential gene expression may identify genes and pathways involved in the process of aneurysm rupture. For example, the lysosome pathway is a new pathway for the rupture of IAs and evidence for the role of the immune response in aneurysmal rupture has been found (Kleinloog et al., 2016). Another study reported that DEGs were mainly enriched in pathways of the major histocompatibility complex class II protein complex and antigen processing and presentation (Wang et al., 2018). We also found that the distinct status of IAs could lead to differential innate immune responses, as neutrophils account for 50–70% of circulating leukocytes and are therefore the most common cells involved in innate immune responses, and they can act as signaling mediators performing various antimicrobial functions and inflammatory responses through activation and degranulation (Klopf et al., 2021). Although some pathways that play a role in the process of IA rupture have been discovered, the key genes that distinguish whether or not rupture occurs have not been elucidated. Using weighted gene co-expression network analysis, Wang et al. (2020) identified six hub genes associated with IA rupture, which tended to enrich in one pathway. In addition, the gene with the highest ranking for classification importance was not necessarily the one with the greatest fold change and vice versa (Li et al., 2022). The feature selection algorithm had the benefit over conventional statistical methods in this regard, favoring the selection of biomarkers that could be used to distinguish between IAs that had ruptured and those that had not. As we were unable to find larger studies investigating gene expression differences in IAs, in the present study, we combined four previous GSE datasets. We propose a new two-stage hybrid feature selection algorithm FCBF-MLP-PSO, which is a mixed filter- and wrapper-based gene selection algorithm. Thereafter, YIPF1, RAB32, WDR62, ANPEP, LRRCC1, AADAC, GZMK, WBP2NL, PBX1, and TOR1B containing a total of 10 effective feature subsets were determined. The highest ACC value increased from 0.817 to 0.919 (12.5% increase), the highest AUC value increased from 0.87 to 0.94 (8.0% increase), and all evaluation metrics improved by approximately 10% after being processed by our proposed gene selection algorithm. By analyzing the relationship of IAs in previous studies with each informative gene, only GZMK was reported to be associated with IA rupture (Wang et al., 2020).

In conclusion, we constructed a novel 10-gene signature (YIPF1, RAB32, WDR62, ANPEP, LRRCC1, AADAC, GZMK, WBP2NL, PBX1, and TOR1B) using the proposed two-stage hybrid algorithm FCBF-MLP-PSO and used different machine learning models to predict the rupture status in IAs (AUC = 0.94, ACC = 0.92, greater than the previous researches, as far as we know). Therefore, these 10 informative genes used to predict the rupture status of IAs can be used as complements to imaging examinations in the clinic, and provide new targets and ideas for the treatment of ruptured IAs.

## Data availability statement

The original contributions presented in this study are included in the article. Further inquiries can be directed to the corresponding author.

## Author contributions

QL and MY conceived and designed the experiments. QL and PW analyzed the data and wrote the first draft of this manuscript. YZ, JY, and YM discussed and contributed to the data analysis. All authors reviewed and approved the final version of the manuscript.

## Footnotes

1
http://www.ncbi.nlm.nih.gov/geo

2
http://timer.cistrome.org/

## Supplementary material

Supplementary Figure 1 The Principal Component Analysis (PCA) plots and the t-Distributed Stochastic Neighbor Embedding (t-SNE) plots before and after adjustment of the batch effects. **(A)** The PCA plots before adjustment of the batch effects, **(B)** the t-SNE plots before adjustment of the batch effects, **(C)** the PCA plots after adjustment of the batch effects, **(D)** the t-SNE plots after adjustment of the batch effects.

Supplementary Figure 2 Importance ranking of the 42 candidate features obtained by the FCBF algorithm.

Supplementary Figure 3 Expression of the selected 10 informative genes across samples in the total dataset and each of the raw datasets between ruptured and unruptured intracranial aneurysms. Violin plots are used to present the total dataset and box plots are used to present individual raw GSE datasets.
