# Supplementary figures and images for: A two-stage hybrid gene selection algorithm combined with machine learning models to predict the rupture status in intracranial aneurysms
Source: Front Neurosci. 2022 Oct 20;16:1034971. doi: 10.3389/fnins.2022.1034971 (PMC9631203; doi:10.3389/fnins.2022.1034971)

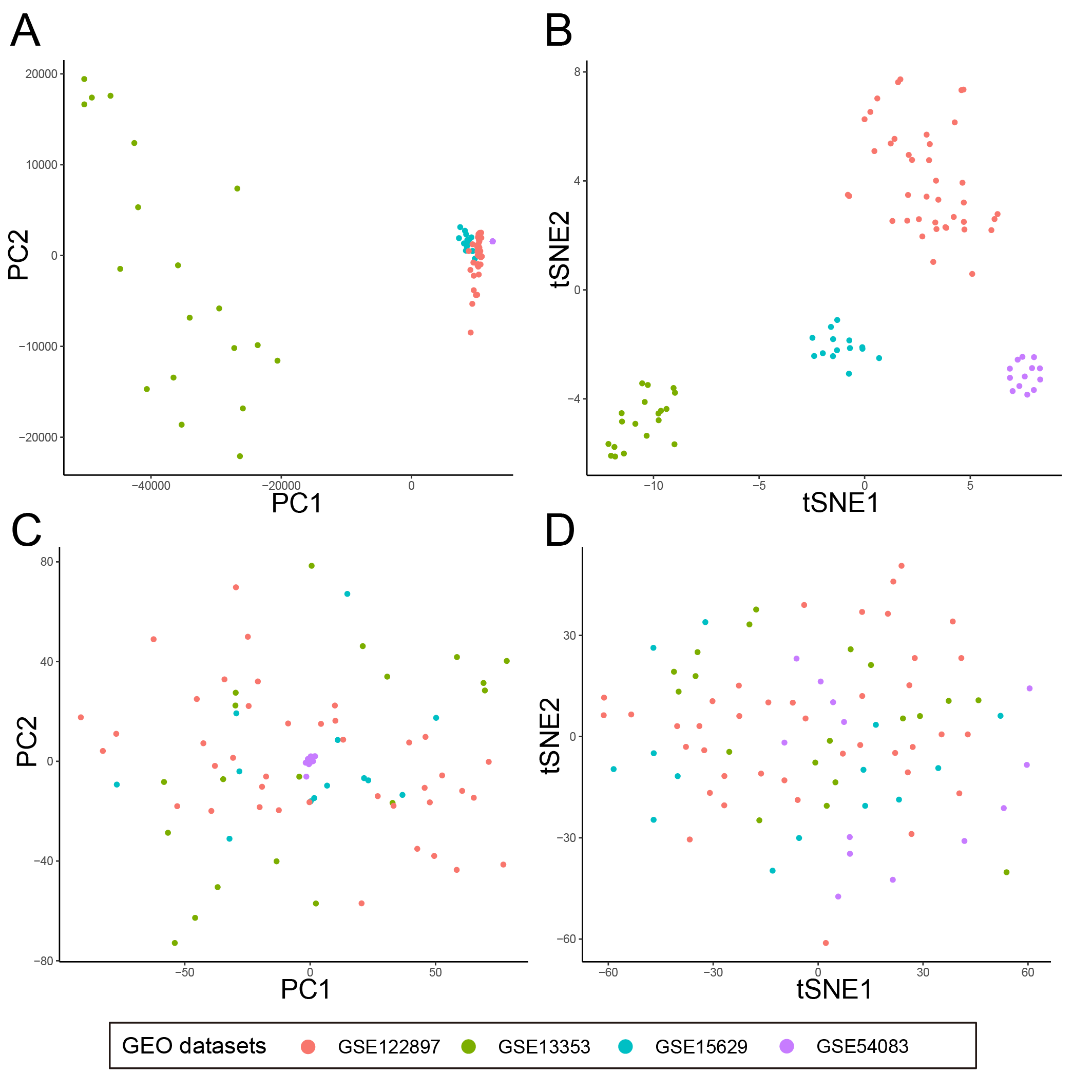

Supplement: Supplementary Figure 1 — The Principal Component Analysis (PCA) plots and the t-Distributed Stochastic Neighbor Embedding (t-SNE) plots before and after adjustment of the batch effects. (A) The PCA plots before adjustment of the batch effects, (B) the t-SNE plots before adjustment of the batch effects, (C) the PCA plots after adjustment of the batch effects, (D) the t-SNE plots after adjustment of the batch effects. [file Image_1.TIF]

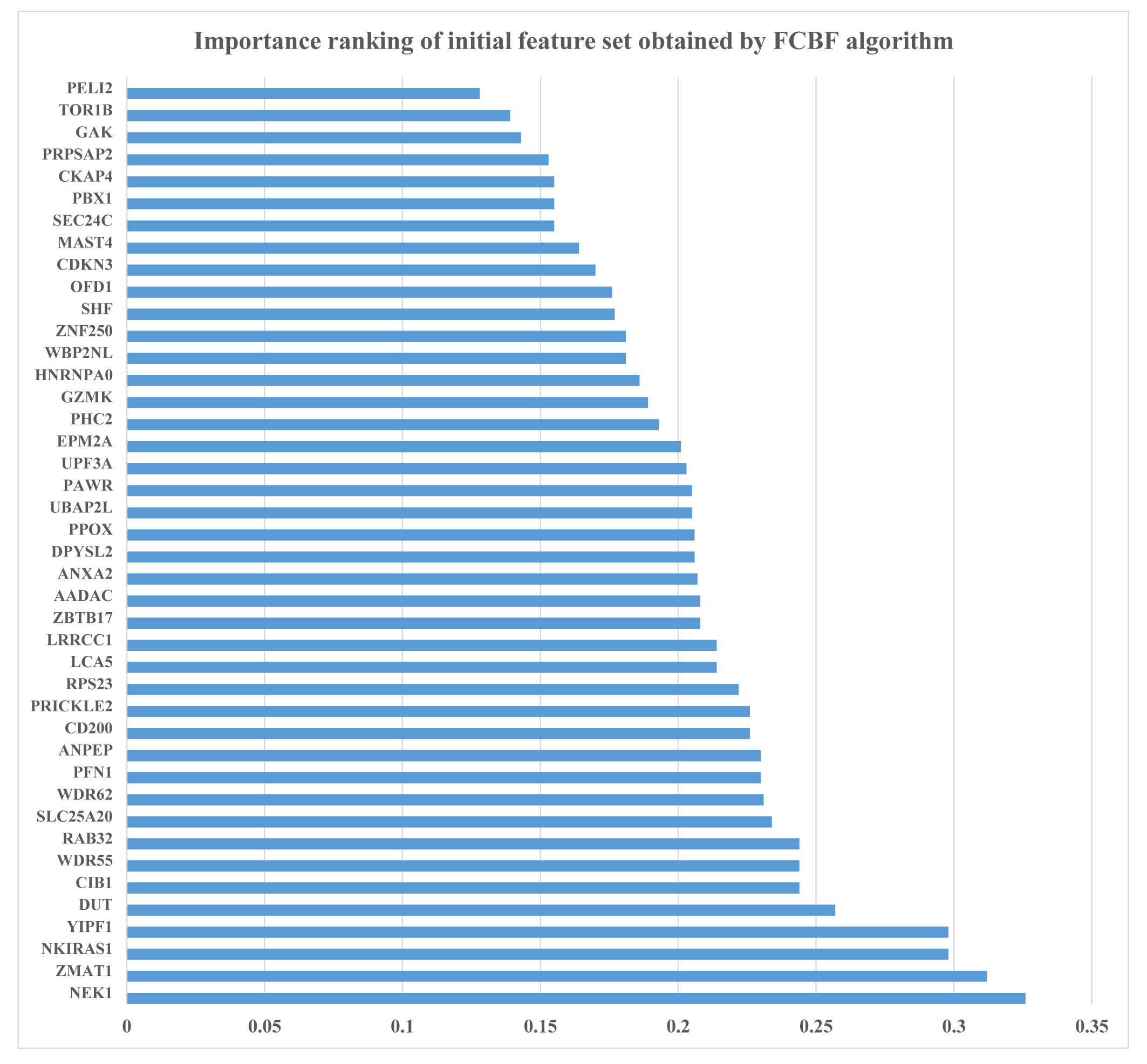

Supplement: Supplementary Figure 2 — Importance ranking of the 42 candidate features obtained by the FCBF algorithm. [file Image_2.TIF]

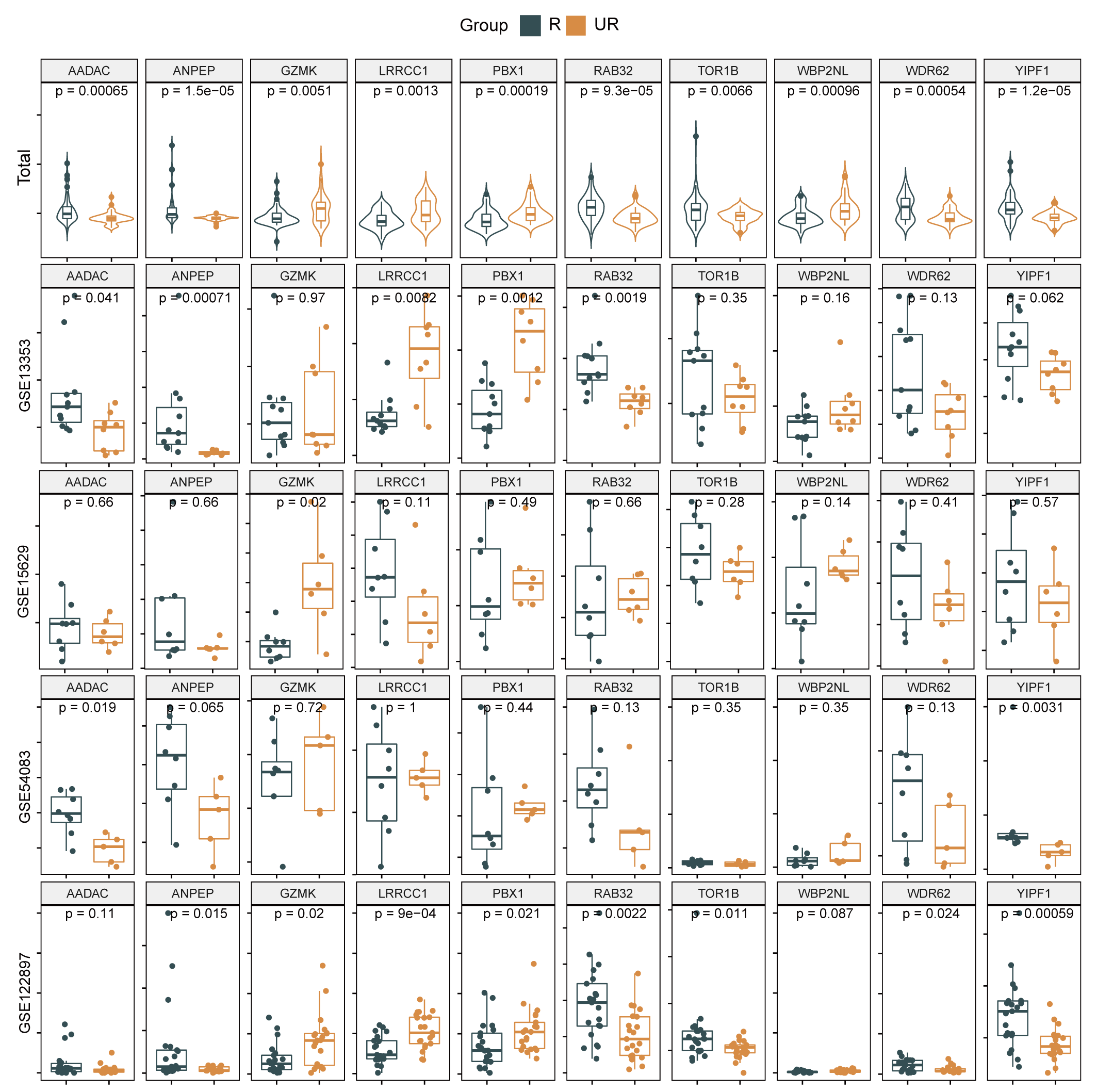

Supplement: Supplementary Figure 3 — Expression of the selected 10 informative genes across samples in the total dataset and each of the raw datasets between ruptured and unruptured intracranial aneurysms. Violin plots are used to present the total dataset and box plots are used to present individual raw GSE datasets. [file Image_3.TIF]
